# Screening of tropical isolates of *Metarhizium anisopliae* for virulence to the red palm weevil *Rhynchophorus ferrugineus* Olivier (Coleoptera: Curculionidae)

**DOI:** 10.1186/s40064-016-2780-6

**Published:** 2016-07-16

**Authors:** Xiaodong Sun, Wei Yan, Weiquan Qin, Jing Zhang, Xiaoqing Niu, Guangchang Ma, Fuheng Li

**Affiliations:** Hainan Key Laboratory of Tropical Oil Crops Biology/Coconuts Research Institute, Chinese Academy of Tropical Agricultural Sciences, Wenchang, 571339 Hainan People’s Republic of China; College of Science, Northeast Agricultural University, Harbin, 150030 Heilongjiang People’s Republic of China; Environment and Plant Protection Research Institute, Chinese Academy of Tropical Agricultural Sciences, Haikou, 571101 Hainan People’s Republic of China

**Keywords:** *Metarhizium anisopliae*, Red palm weevil, ITS, Mortality, Bioassay

## Abstract

The red palm weevil, *Rhynchophorus ferrugineus* Olivier (Coleoptera: Curculionidae) is a serious pest of the palm tree in tropical regions of the world. One strain of *Metarhizium* sp. ZJ-1, isolated from Chinese soils, was evaluated for growth characteristics, and screened for its virulence to *R. ferrugineus* larvae in laboratory conditions. An approximately 685-bp fragment was amplified by ITS (ITS1-5.8S-ITS2) PCR from strain ZJ-1, further phylogenetic analysis revealed that 93 % similarity to *Metarhizium anisopliae.* Inoculation of 1 × 10^8^ conidia/mL caused 100 % mortality of *R. ferrugineus*, LT50 levels of ZJ-1 were 1.66 days (1 × 10^8^ conidia/mL), indicating that the conidia of strain ZJ-1 were highly virulent. These results suggest that *M. anisopliae* ZJ-1 has potential as an effective and persistent biological control agent for *R. ferrugineus*.

## Background

Invasive red palm weevil, *Rhynchophorus ferrugineus* Olivier (Coleoptera: Curculionidae), is one of the most severe pests of various palm species (Tagliavia et al. 2014). It was first observed in India, but now is widely distributed in Asia, Africa, Central America, South America, and the Caribbean, as well as in Curaçao, Netherlands Antilles (Fiaboe and Roda 2012; Kehat 1999).

In China, *R. ferrugineus*is was considered as quarantine pest and has 19 species of 15 palm genera (Kehat 1999; Dembilio et al. 2009). According to the international standards for pest measurements (ISPM), the results from an *R. ferrugineus* pest risk analysis model showed that its evaluation is close to that of a high-level quarantine pest. This indicates that *R. ferrugineus* can easily settle in China, where it potentially poses a great threat to palm trees (Wu et al. 2007).

*Rhynchophorus ferrugineus* can spread within tree trunks, destroy the vascular system, eventually, the trees soon died (Fetoh 2011). Female *R. ferrugineus* lay eggs in injured trunks of established palms, associated frond bases, tree crowns, and offshoots. *R. ferrugineus* larvae bore into palm crowns, trunks, and offshoots and are generally invisible until the infected palms are dying (Pinhas et al. 2008). According to integrated pest management strategy, several control methods have been used against *R. ferrugineus* invasion of palms. Some of these methods include cutting down and burning infected palms, trapping adult *R. ferrugineus*, chemical control, host plant resistance, and male sterile techniques (Faleiro 2006). Aside from these, numerous entomopathogenic fungi (*Metarhizium anisopliae* and *Beauveria abassiana*) isolated from *R. ferrugineus* in the Middle East have been evaluated for biocontrol of associated pest insects (Gindin et al. 2006). However, biocontrol has not been widely used, although it is friendly to the environment and harmless to humans. Despite the economic importance of investigations of infection patterns and histopathology of these entomopathogenic fungi in selected insects, no study has documented the histopathology of entomopathogenic fungi in *R. ferrugineus* (Toledo et al. 2010).

A new Chinese variety of *M. anisopliae* ZJ-1, characterized by high virulence and low repellency to *R. ferrugineus*, was obtained. In the present study, we sequenced the ITS1-5.8S-ITS2 region of *M. anisopliae* strain ZJ-1 and identified *M. anisopliae* within infected *R. ferrugineus* larvae.

## Results

### Fungal morphology of fungi

Sporulation color in *Metarhizium* can be light to very dark green (Bischoff et al. 2006), and while green sporulation is characteristic of *Metarhizium* spp., it cannot be considered as diagnostic, because other fungi also produce green spores. The morphology of strain ZJ-1 was typical of the genus *M. anisopliae*. It grew fast on SDAY and PDA plates. Round colonies on agar plates were white or cream, with villous and gossypine aerial hyphae on the surface. Punctiform olive green formed in the middle of the colony, which turned darker during growth and resulted in a tawny color at the back of the plates.

The morphology of hyphae and conidial of *M. anisopliae* ZJ-1 from *R. ferrugineus* cadavers were showed. The mycelium was smooth and hyaline, well-branched and separated, with major hyphae up to 2.0–3.2 μm wide. Conidiophores were podgy, simple, or highly branched, with 2–5 sterigma at the branch top. Conidia were colorless, elliptical, and cylindrical, with obtuse ends. Most conidia were 4.8–5.5 μm × 2.0–2.5 μmin length. Growth temperatures were between 10 and 38 °C, with an optimum of 28 °C.

### Molecular identification

An approximately 685 bp fragment was amplified by ITS (ITS1-5.8S-ITS2) PCR from strain ZJ-1. The ITS sequence was submitted to GenBank (accession No. JN377427). The ZJ-1 sequence bore 93 % similarity to that of *M. anisopliae* (FJ545294) according to an NCBI Blast search. This result indicated that strain ZJ-1 could represented a new species and possibly a member of a new genus.

**Fig. 1:**
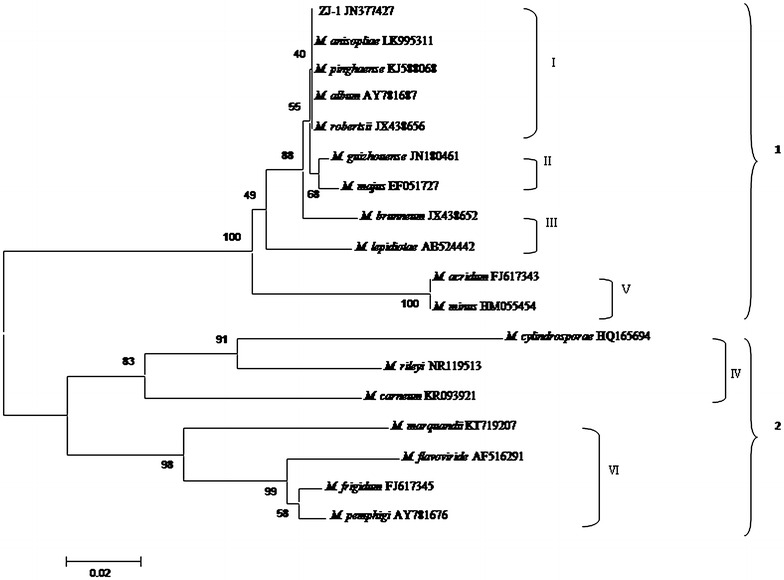
The clustering analysis tree of sample strains and 17 kinds of *Metarhizium* ITS sequences

The dendrogram bifurcated into two distinct clades (1 and 2) with a dissimilarity of 0.02 (Fig. 1). Clade 1 consists of four additional clusters, in which isolates ZJ-1, *M. anisopliae*, *M. pinghaense*, *M. album*, *and M. robertsii* are identical (cluster I); *M. guizhouense*, *M. majus*, *M. acridum*, and *M. minus* show a high similarity (cluster II, cluster IV); and cluster IV separates from the other clusters. Clade 2 is represented by two large clusters (V and VI); these sequences show the lowest similarity to the ITS sequences of the isolate ZJ-1 used in this investigation. The origin of 17 kinds of *Metarhizium* ITS sequences are shown in Table 1.

**Table 1:** Origin of 17 kinds of *Metarhizium* ITS sequences

Name	Location	Bankit	Name	Location	Bankit
*Metarhizium pinghaense*	Italy	KJ588068	*Metarhizium anisopliae*	Algeria	LK995311
*Metarhizium album*	Brazil	AY781687	*Metarhizium robertsii*	Poland	JX438656
*Metarhizium guizhouense*	China	JN180461	*Metarhizium majus*	Brasil	EF051727
*Metarhizium brunneum*	Poland	JX438652	*Metarhizium pemphigi*	Brazil	AY781676
*Metarhizium lepidiotae*	Japan	AB524442	*Metarhizium acridum*	USA	FJ617343
*Metarhizium frigidum*	USA	FJ617345	*Metarhizium minus*	China	HM055454
*Metarhizium cylindrosporae*	Thailand	HQ165694	*Metarhizium rileyi*	USA	NR119513
*Metarhizium carneum*	Brazil	KR093921	*Metarhizium marquandii*	China	KT719207
*Metarhizium flavoviride*	Greece	AF516291			

### Laboratory bioassays

**Fig. 2:**
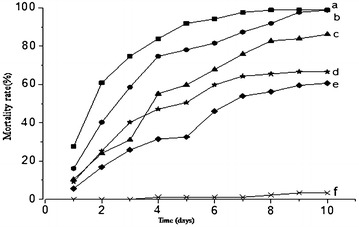
Mortality rates of *R. ferrugineus* larvae treated with different doses of *M. anisopliae.* Dose of Conidia/mL: *cross symbol* control; *filled square* 1 × 10^8^; *filled circle* 1 × 10^7^; *filled triangle* 1 × 10^6^; *filled star* 1 × 10^5^; *filled diamond* 1 × 10^4^. *a*–*e* *Different letters* indicate statistically significant differences (P < 0.05)

Results showed that conidia of *M. anisopliae* ZJ-1 killed 60 % of test specimens at the lowest concentration (1 × 10^6^ conidia/mL) 10 days post-inoculation. The mortality rate increased with conidial concentration (*P* < 0.05). Conidia were found to be highly virulent to *R. ferrugineus* and caused approximately 100 % mortality 8 days post-inoculation of 1 × 10^8^ conidia/mL conidia. In contrast, the control group showed significantly lower mortality rates (3.33 ± 2.87 %) (Fig. 2). LT50 values for *M. anisopliae* ZJ-1 were 1.66 day (1 × 10^8^ conidia/mL) (Table 2). Probit analysis showed that LD50 values were 2175.29 conidia/larvae 3-day post-treatment.

**Table 2:** LT50 values of *R. ferrugienus* infected by *M. anisopliae* isolates (95 % CL)

Spores concentration	LT50 (day)	Correlation coefficient R	95 % CL	
			Lower	Upper
1 × 10^8^	1.66	0.9967	1.46	1.87
1 × 10^7^	2.44	0.9969	2.20	2.70
1 × 10^6^	3.78	0.9764	3.46	4.12
1 × 10^5^	4.73	0.9923	4.23	5.31
1 × 10^4^	7.14	0.9810	6.37	8.01

*LT50*, lethal time for 50 % mortality

## Discussion

This study showed that entomopathogenic fungus *M. anisopliae* ZJ-1 was highly and stably virulent to *R. ferrugineus* LT50, ZJ-1 killing the treated larvae rather quickly (LT50:2.44d) compared with *M. anisopliae* isolate (LT50:3.5) (Gindin et al. 2006). It was a serious pest of various palm species. biological control with pathogenic fungi can offer long-terminsect control without destroying environment and non-target organisms(Rossetti et al. 2015). The pathogenic fungi seem (easy) to survive and distribute with *R. ferrugineus* in dark and humid surroundings. It is generally known that the studies in China describing the biological control of *R. ferrugienus* with entomopathogenic fungi have not yet been reported.

Driver et al. (2000) analyzed ITS1 and ITS2 sequences of 100 representative *M. anisopliae* strains and found ITS to be a good basis for the classification of *M. anisopliae*. Islam et al. (2014) sequenced the internal transcribed spacer (ITS) 1-5.8-ITS4 region of various *M. anisopliae* strains for the detection and identification of *M. anisopliae.* In the present study, we determined rDNA-ITS sequences of *M. anisopliae* ZJ-1 and analyzed associated sequences of relevant varieties. Results indicated isolate ZJ-1 to be a novel variety of *M. anisopliae*, with 93 % sequence identity to *M. anisopliae*, which was consistent with results from morphological characterization. *M. anisopliae* is applied as conidia or mycelia in various formulations. The new conidia and viable cells can be spread to the healthy insects and thus achieve the control effect through infecting the insects and the induction of a fungal epizootic (Genthner et al. 1997). After the series of adhesion, prepenetration growth, penetration into the host, and establishment of the pathogen in the host, the insects would be infected by *M. anisopliae*.

## Conclusions

In summary, this study showed high susceptibility of *R. ferrugienus* larva to certain concentrations of local *M. anisopliae* ZJ-1. Future application of *M. anisopliae* ZJ-1 in the field is recommended for in situ protection of local palm trees. However, the new delivery methods along with more persistent formulations for this fungus are necessary to improve its efficiency and persistence under field conditions in the future.

## Methods

### Entomopathogenic fungus

Naturally dead *R. ferrugineus* cadavers were collected from Wenchang, Hainan Island, China (19_32N, 110_47E). The samples were soaked in 70 % alcohol for 1 min and rinsed using sterile distilled water. The cadavers were subsequently surface-sterilized using 0.1 % mercury chloride, followed by three rinses in sterile distilled water. Parts of the tissues were cut and inoculated on Sabouraud Dextrose Agar with Yeast Extract (SDAY) containing 40 g/L dextrose, 10 g/L peptone, 10 g/L yeast, 20 g/L agar and 500 µg/mL streptomycin. These tissues were separately placed on sterile petri dishes sealed with preservative film at 28 ± 1 °C, 75 ± 5 % RH for 6 days. Purification was achieved using a monospore culture, named ZJ-1. Scanning electron microscopy (Hitachi S-3000N) was performed to study morphologic characteristics of ZJ-1 (Driver et al. 2000; Tulloch 1976; Su 2006).

### Experimental insects

A laboratory population of *R. ferrugineus* was established by collecting larvae from infected palm trees in the Wenchang suburb (19_32N, 110_47E), Hainan, China (Li et al. 2010). Larvae were reared on sugarcane stem tissues at 28 ± 1 °C, 75 ± 5 % RH. After adults emerged, they were placed in jars and supplied with cotton wicks saturated with 8–10 % honey for feeding. Subsequently, eggs were transferred to a moist sterile filter paper within an unsealed petri dish (12 cm in diameter). Upon hatching, neonate larvae were individually transferred to 50 mL vials containing 10 g weevil’s artificial diet (Martín and Cabello 2006). Approximately 7 days later, laboratory-reared larvae were obtained for further analysis.

### DNA extraction and sequencing

Genomic DNA (gDNA) was extracted following the method described by Lee and Taylor (Lee and Taylor 1990). A fragment of the ITS spacer region was amplified using universal primer sets ITS4 (5′-TCCTCCGCTTATTGATATGC-3′) and ITS5 (5′-GGAAGTAAAAGTCGTAACAAGG-3′). PCR reactions (50 µL) contained 50 ng of template gDNA, 1 µL of each 10 pM oligonucleotide, 1 µL of 10 mM dNTPs, 1 µL of 2 U/µL *Taq* DNA polymerase (Sino Geno Max Co., Ltd, Beijing), 5 µL of 10× PCR buffer. The PCR protocol for amplification of ITS regions included initial denaturation at 94 °C for 5 min, 35 cycles at 94 °C for 30 s, 55 °C for 30 s, and 72 °C for 30 s, followed by a final elongation at 72 °C for 10 min. PCR products were kept at 4 °C. The size and quality of PCR products were determined by gel electrophoresis using 1.25 % agarose gel, which was stained with ethidium bromide (0.5 mg/mL) and visualized under UV light. The ITS1-5.8S-ITS2 amplified products were purified using a Fungus Genomic DNA Extraction Kit (Omega) and sequenced in an automated system (Sino Geno Max Co., Ltd, Beijing).

DNA sequences obtained from this work were compared with existing 17 *Metarhizium* species sequences data in GenBank using the Basic Local Alignment Search Tool (NCBI BLAST) (Destéfano 2004); a summary of these comparisons is shown in Table 1. Phylogenetic relationships among DNA sequences of ZJ-1 were determined using multiple sequence alignment MAGA6.0 via the maximum likelihood method (ML). Confidence levels for generated groups were determined via 1000-repetition bootstrap analysis.

### Laboratory bioassays

We selected *R. ferrugineus* larvae of similar size during the feeding period. Conidia of divided purified ZJ-1 were placed in a sterile 10 mL centrifuge tube containing aqueous 0.05 % Tween-80 and the mixture was vortexed to attain homogenization. Conidial concentration was determined using a hemocy to meter. A dilution series of aqueous conidial suspension (1.0 × 10^8^, 1.0 × 10^7^, 1.0 × 10^6^, 2.0 × 10^5^, 1 × 10^4^ conidia/mL) was prepared thorough mixing, then sprayed on larvae. Treated larvae were separately transferred to 50 mL vials containing 10 g weevil’s artificial diet under controlled conditions (28 ± 1 °C, 80 ± 5 % RH). Each trial was performed in triplicate with a total of 30 insects per process. After the treatment, insects were scored as dead at 24 h intervals for 10 days. Meanwhile, dead larvae were selected and tested for potential pathological changes (Anggraeni and Putra 2011).

## Abbreviations

*M. anisopliae*: *Metarhizium anisopliae*
*B. abassiana*: *Beauveri abassiana*
RPW: red palm weevil
*R. ferrugineus*: *Rhynchophorus ferrugineus* Olivier (Coleoptera: Curculionidae)
ITS: internal transcribed spacer
SDAY: Sabouraud dextrose agar with yeast extract
